# Supplementary material for: New Chlorinated Metabolites and Antiproliferative Polyketone from the Mangrove Sediments-Derived Fungus Mollisia sp. SCSIO41409
Source: Mar Drugs. 2022 Dec 30;21(1):32. doi: 10.3390/md21010032 (PMC9866852; doi:10.3390/md21010032)
Supplement: Supplementary file 1 [file marinedrugs-21-00032-s001.zip › marinedrugs-2108902-supplementary.pdf]

Supporting Information

New Chlorinated Metabolites and Antiproliferative Polyketone from the Mangrove Sediments-Derived Fungus *Mollisia* sp. SCSIO41409

Jian Cai [1,2,†], Xueni Wang [1,3,†], Xia Gan [3,4], Qian Zhou [3], Xiaowei Luo [4], Bin Yang [1,2], Yonghong Liu [1,2], Disna Ratnasekera [5,*] and Xuefeng Zhou [1,2,*]

[1] CAS Key Laboratory of Tropical Marine Bio-resources and Ecology, Guangdong Key Laboratory of Marine Materia Medica, South China Sea Institute of Oceanology, Chinese Academy of Sciences, Guangzhou 510301, China

[2] University of Chinese Academy of Sciences, Beijing 100049, China

[3] Guangxi Zhuang Yao Medicine Center of Engineering and Technology, Guangxi University of Chinese Medicine, Nanning 530200, China

[4] Institute of Marine Drugs, Guangxi University of Chinese Medicine, Nanning 530200, China

[5] Department of Agricultural Biology, Faculty of Agriculture, University of Ruhuna, 81000, Sri Lanka

* Correspondence: disnar@agbio.ruh.ac.lk (D.R.); xfzhou@scsio.ac.cn (X.Z.)

† These authors contributed equally to this work.

Table of Contents

The physicochemical data of the known compounds 2 and 4–9 3

Figure S1: ^{1}H NMR spectrum of **1** in DMSO-d_6.. 5

Figure S2: ^{13}C NMR spectrum of **1** in DMSO-d_6... 5

Figure S3: DEPT135 spectrum of **1** in DMSO-d_6. 6

Figure S4: HSQC spectrum of **1** in DMSO-d_6. 6

Figure S5: HMBC spectrum of **1** in DMSO-d_6. 7

Figure S6: ^{1}H–^{1}H COSY spectrum of **1** in DMSO-d_6. 7

Figure S7: HRESIMS spectrum of **1**.. 8

Figure S8: IR spectrum of **1**. 8

Figure S9: UV spectrum of **1** in MeOH. 9

Figure S10: ^{1}H NMR spectrum of **3** in DMSO-d_6.. 9

Figure S11: ^{13}C NMR spectrum of **3** in DMSO-d_6... 10

Figure S12: HRESIMS spectrum of **3**. 10

Figure S13: IR spectrum of **3**. 11

Figure S14: UV spectrum of **3** in MeOH. 11

ITS sequence of the strain *Mollisia sp*. SCSIO41409... 12

The physicochemical data of the known compounds 2 and 4–9

5-hydroxy-2,3-dimethyl-7-methoxychromone (**2**): white needles; ^{1}H NMR (500 MHz, DMSO-d_6) δ 13.00 (s, 1H, 5-OH), 6.53 (d, J = 2.3 Hz, 1H, H-6), 6.33 (d, J = 2.3 Hz, 1H, H-8), 3.83 (s, 3H, H-11), 2.39 (s, 3H, H-9), 1.91 (s, 3H, H-10); ^{13}C NMR (125 MHz, DMSO) δ 181.7 (C-4), 165.4 (C-5), 164.2 (C-2), 161.6 (C-5), 157.6 (C-8a), 114.9 (C-3), 104.2 (C-4a), 98.1 (C-6), 92.3 (C-8), 56.5 (C-11), 18.8 (C-9), 9.3 (C-10).

Stemphone C (**4**): yellow needles; HRESIMS *m/z* 515.3003 $[M+H]^+$ (calcd $C_{30}H_{43}O_7$, 515.3003); $[\alpha]_D^{25}$ +160.9 (c 0.01, CH_3OH); ^{1}H NMR (500 MHz, DMSO-d_6) δ 6.59 (s, 1H, H-11), 5.52 (q, J = 6.5 Hz, 1H, H-2), 5.16 (d, J = 9.2 Hz, 1H, H-4), 4.14 (s, 1H, 22-OH), 3.21 (dd, J = 9.1, 6.9 Hz, 1H, H-21), 3.17 (m, 1H, overlapped, H-21), 3.08 (dd, J = 11.4, 3.5 Hz, 1H, H-17), 2.36 (dd, J = 18.4, 4.6 Hz, 1H, H-12a), 2.09(m, 1H, H-12b), 2.0(m, 3H, H-15, 19a), 1.86 (s, 3H, H-30), 1.73 (m, 2H, H-16), 1.57 (s, 3H, H-28), 1.56 (d, J = 4.0 Hz, 3H, H-1), 1.45 (m, 3H, overlapped, H-13, 20), 1.24 (m, 1H, H-19b), 1.19 (s, 3H, H-26), 1.08 (s, 3H, H-24), 1.04 (s, 3H, H-23), 0.95 (d, J = 7.1 Hz, 3H, H-27), 0.81 (s, 3H, H-25); ^{13}C NMR (125 MHz, DMSO) δ 186.5 (C-10), 180.9 (C-7), 169.3 (C-29), 151.7 (C-8), 147.3 (C-6), 132.3 (C-11), 132.1 (C-3), 124.5 (C-2), 117.6 (C-9), 84.8 (C-21), 83.2 (C-17), 81.0 (C-4), 80.2 (C-14), 70.3 (C-22), 45.7 (C-13), 36.8 (C-15), 36.0 (C-19), 35.0 (C-18), 33.1 (C-5), 26.9 (C-24), 25.0 (C-16), 24.6 (C-23), 20.7 (C-30), 20.5 (C-20), 20.5 (C-26), 16.5 (C-27), 16.0 (C-12), 12.9 (C-1), 11.9 (C-25), 11.2 (C-28).

Cis-cyclo (Tyr-Ile) (**5**) : white powder; ^{1}H NMR (500 MHz, DMSO-d_6) δ 9.15 (s, 1H, 11-OH), 8.03 (s, 1H, NH-1), 7.86 (s, 1H, NH-4), 6.95 (d, J = 8.4 Hz, 2H, H-9, 13), 6.61 (d, J = 8.4 Hz, 2H, H-10, 12), 4.11 (t, J = 4.1 Hz, 1H, H-3), 3.55 (s, 1H, H-6), 3.04 (dd, J = 13.7, 4.0 Hz, 1H, H-7a), 2.73 (dd, J = 13.7, 4.8 Hz, 1H, H-7b), 1.41 (m, 1H, H-14), 0.60 (d, J = 6.8 Hz, 1H, H-17), 0.59 (t, J = 7.0 Hz, 3H, H-16). ^{13}C NMR (125 MHz, DMSO) δ 166.5 (C-2), 166.4 (C-5), 156.3 (C-11), 131.3 (C-9), 131.3 (C-13), 126.2 (C-8), 114.7 (C-10), 114.7 (C-12), 58.9 (C-3), 55.3 (C-6), 37.9 (C-7), 36.9 (C-14), 23.1 (C-15), 14.6 (C-17), 11.7 (C-16).

4,8-dihydroxy-1-tetra-lone (**6**): white solid; ^{1}H NMR (500 MHz, DMSO-d_6) δ 12.41 (s, 1H, 8-OH), 7.55 (t, J = 8.0 Hz, 1H, H-6), 7.08 (d, J = 7.5 Hz, 1H, H-5), 6.85 (dd, J = 8.5, 1.1 Hz, 1H, H-7), 4.75 (dt, J = 9.4, 4.8 Hz, 1H, H-4), 2.74 (m, 2H, H-2), 2.19 (m, 1H, H-3); ^{13}C NMR (125 MHz, DMSO) δ 205.3 (C-1), 161.5 (C-8), 148.7 (C-4a), 136.8 (C-6), 117.4 (C-7), 115.9 (C-1a), 115.1 (C-8a), 66.0 (C-

4), 35.3 (C-2), 31.5 (C-3).

Cyclo (Phe-Tyr) (**7**): white powder; ^{1}H NMR (500 MHz, DMSO-d_6) δ 9.22 (1H, s, 11-OH); 7.85 (s, NH-1, 4), 7.27 (m, J = 7.5 Hz, 2H, H-17, 19), 7.20 (t, J = 7.3 Hz, 1H, H-18), 7.03 (d, J = 7.0 Hz, 2H, H-16, 20), 6.83 (d, J = 8.4 Hz, 2H, H-9, 13), 6.67 (d, J = 8.4 Hz, 2H, H-10, 12), 3.95 (m, 1H, H-3), 3.89 (d, J = 6.3 Hz, 1H, H-6), 2.18 (m, 2H, H-7); ^{13}C NMR (125 MHz, DMSO) δ 166.3 (C-2), 166.2 (C-5), 156.1 (C-11), 136.7 (C-15), 130.9 (C-9, 13), 129.8 (C-17, 19), 128.2 (C-16, 20), 126.5 (C-8), 126.4 (C-18) 115.0 (C-10, 12) 55.7 (C-3), 55.4 (C-6), 39.2 (C-14), 38.5 (C-7).

Tenuissimasatin (**8**): white solid; $[\alpha]_D^{25}$ −15.7 (c 0.01, CH_3OH); ^{1}H NMR (500 MHz, DMSO-d_6) δ 7.52 (t, J = 7.8 Hz, 1H, H-6), 7.01 (d, J = 7.4 Hz, 1H, H-7), 6.90 (d, J = 8.1 Hz, 1H, H-5), 5.72 (dd, J = 8.5, 4.0 Hz, 1H, H-3), 3.64 (s, 3H, H-12), 3.19 (dd, J = 16.6, 3.9 Hz, 1H, H-4a), 2.76 (dd, J = 16.6, 8.5 Hz, 1H, H-4b); ^{13}C NMR (125 MHz, DMSO) δ 169.9 (C-1), 167.6 (C-11), 156.9 (C-8), 151.1 (C-10), 136.1 (C-6), 115.9 (C-7), 112.4 (C-5), 111.2 (C-9), 75.7 (C-3), 51.7 (C-12), 38.7 (C-4).

4-methyl-5,6-dihydro-2H-pyran-2-one (**9**): colorless oil; ^{1}H NMR (500 MHz, DMSO-d_6) δ 5.74 (d, J = 1.5 Hz, 1H, H-3), 4.29 (t, J = 6.2 Hz, 2H, H-6), 2.37 (t, J = 6.4 Hz, 2H, H-5), 1.96 (d, J = 1.3 Hz, 3H, H-7); ^{13}C NMR (125 MHz, DMSO) δ 164.0 (C-2), 159.7 (C-4), 115.5 (C-3), 65.7 (C-6), 28.6 (C-5), 22.5 (C-7).

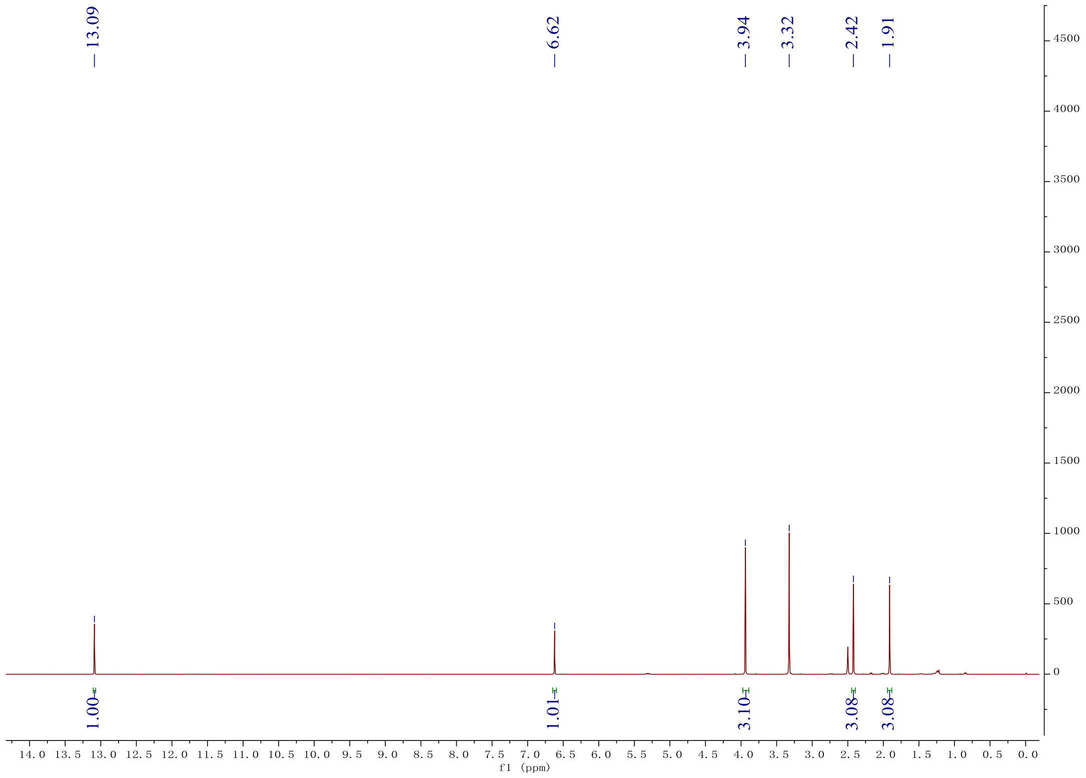

Figure S1: ^{1}H NMR spectrum of **1** in DMSO-d_6.

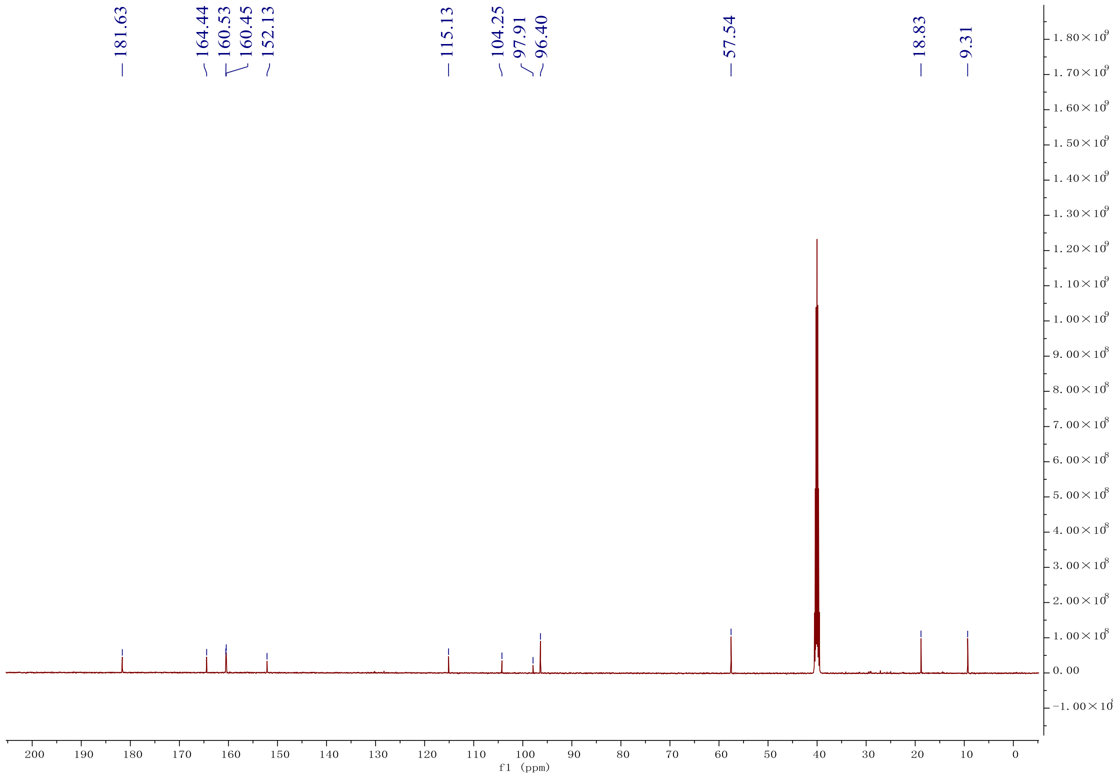

Figure S2: ^{13}C NMR spectrum of **1** in DMSO-d_6.

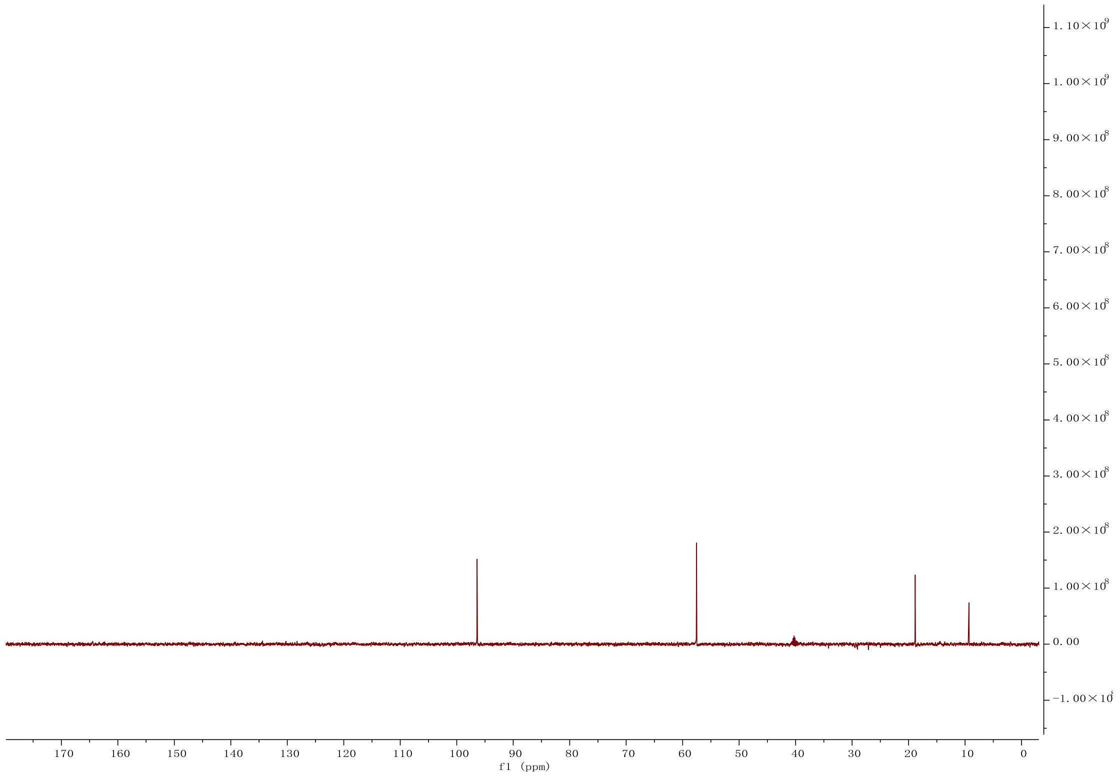

Figure S3: DEPT135 spectrum of **1** in DMSO-d_6.

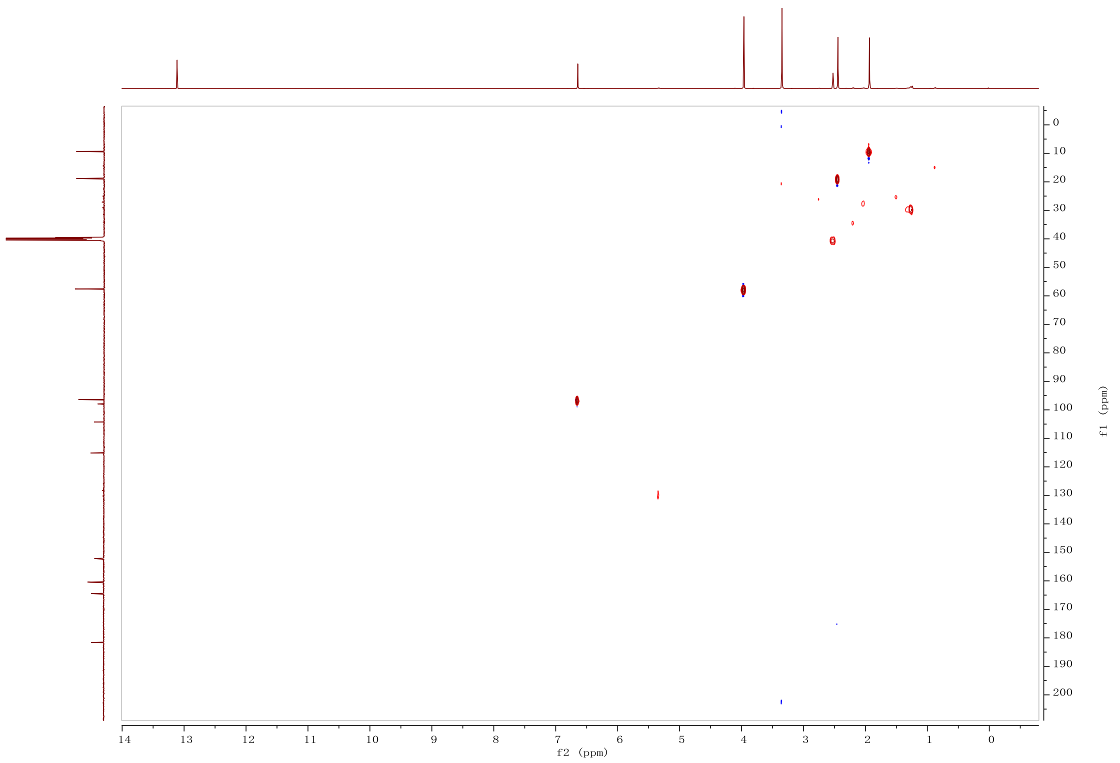

Figure S4: HSQC spectrum of **1** in DMSO-d_6.

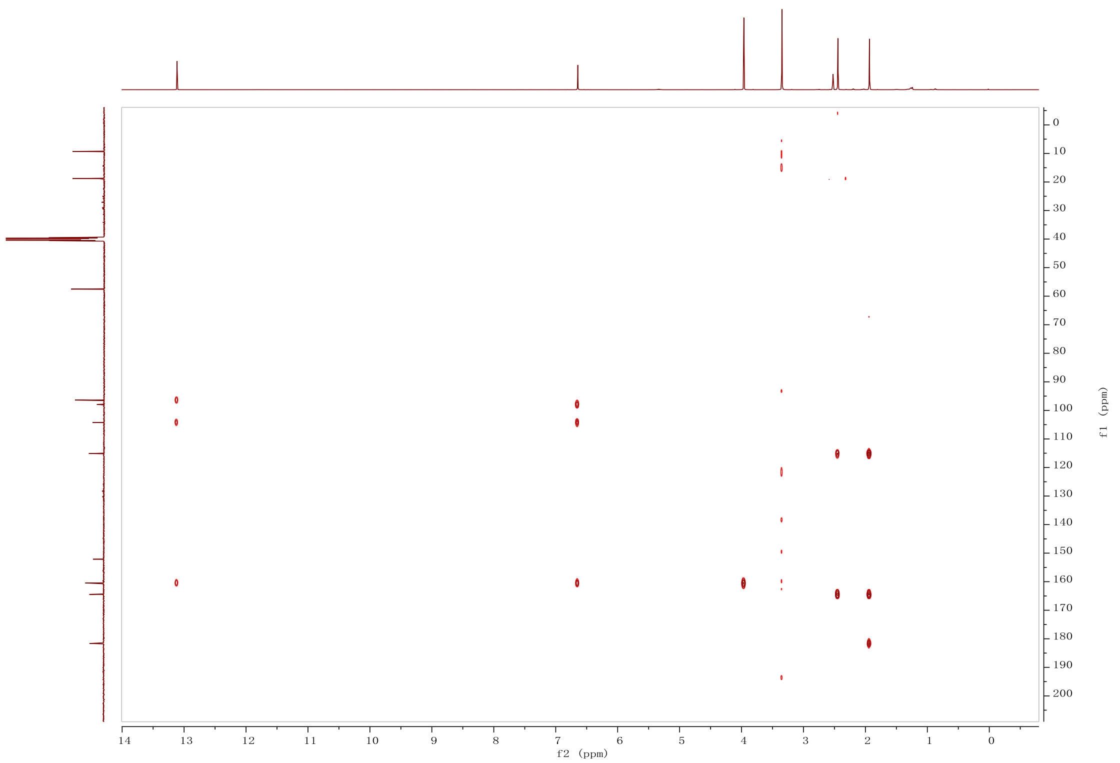

Figure S5: HMBC spectrum of **1** in DMSO-d_6.

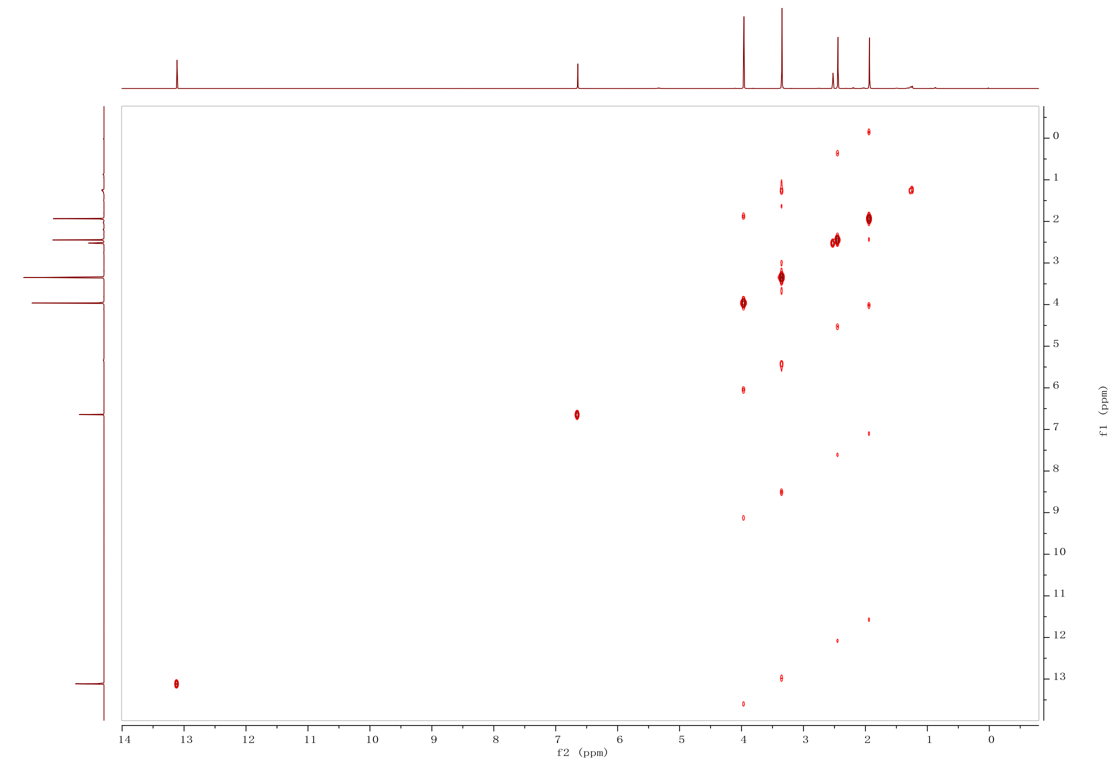

Figure S6: ^{1}H–^{1}H COSY spectrum of **1** in DMSO-d_6.

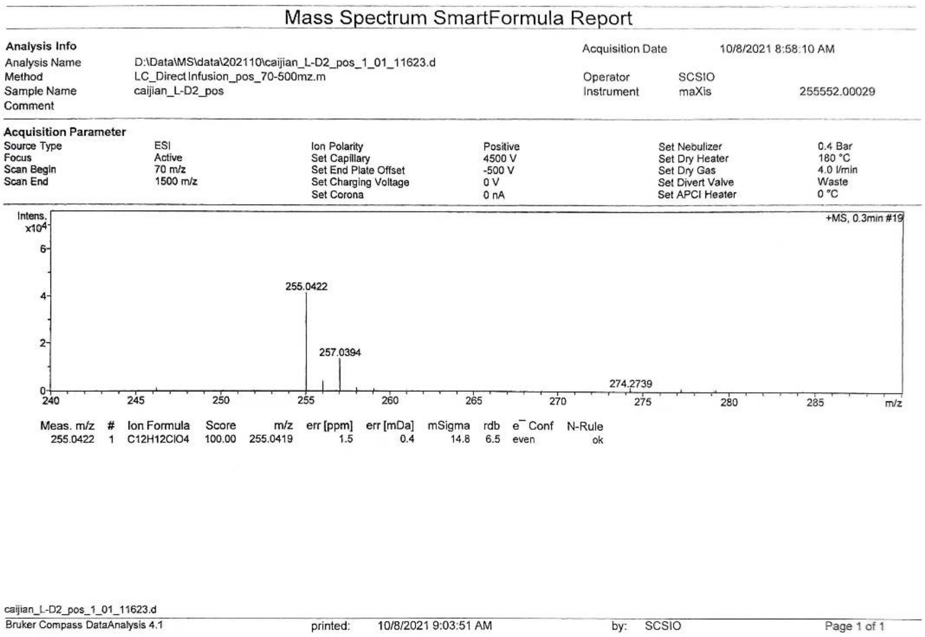

Figure S7: HRESIMS spectrum of **1**.

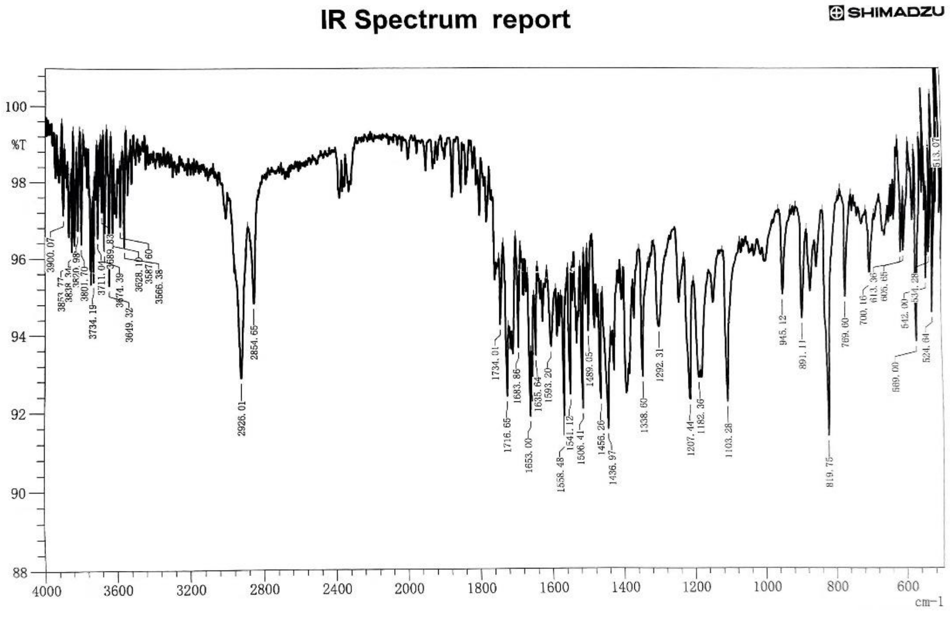

Figure S8: IR spectrum of **1**.

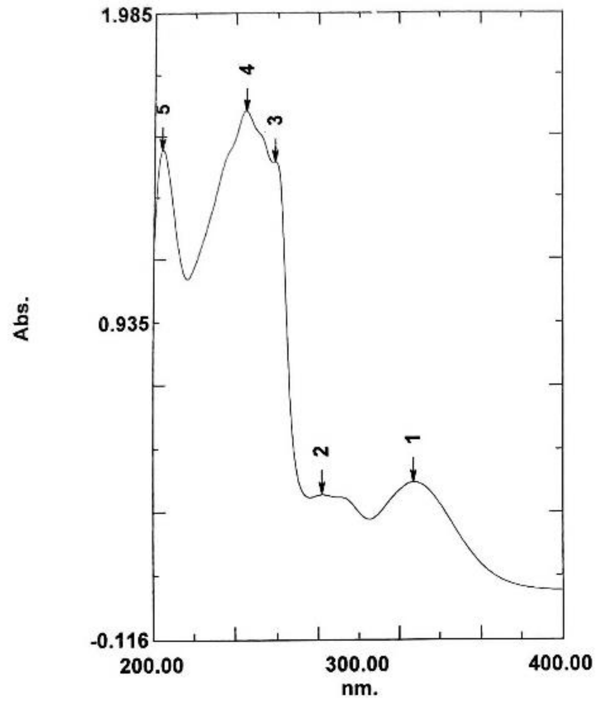

Figure S9: UV spectrum of **1** in MeOH.

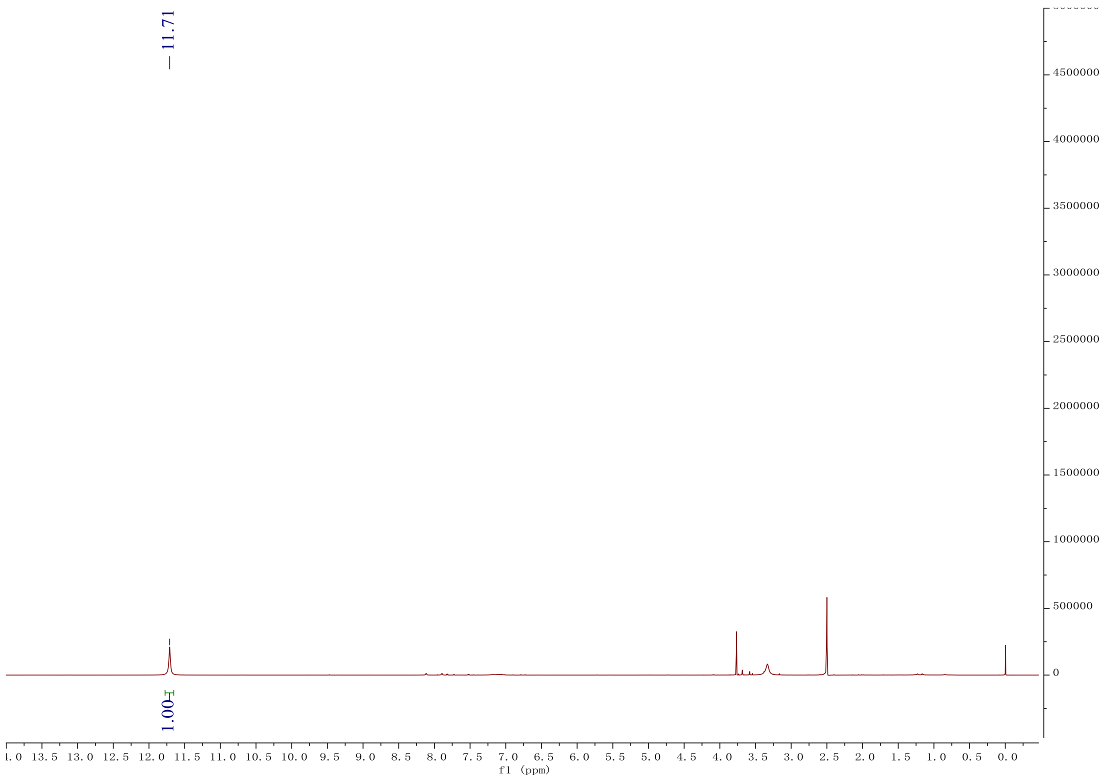

Figure S10: ^{1}H NMR spectrum of **3** in DMSO-d_6.

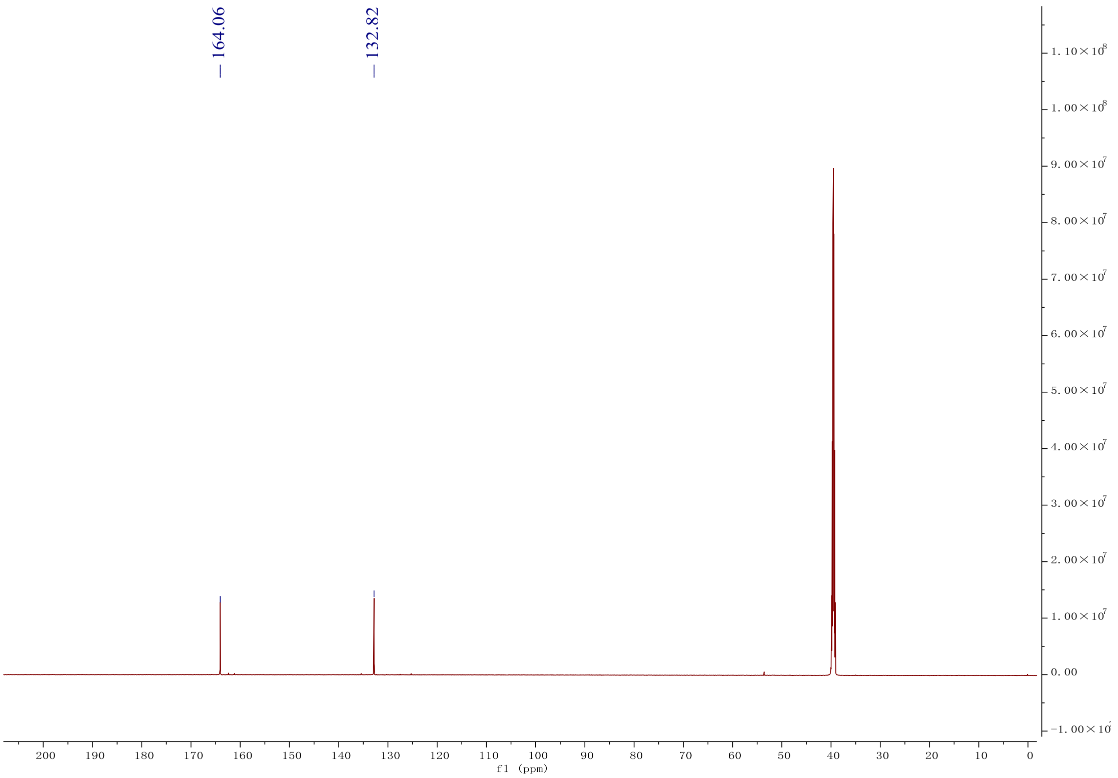

Figure S11: ^{13}C NMR spectrum of **3** in DMSO-d_6.

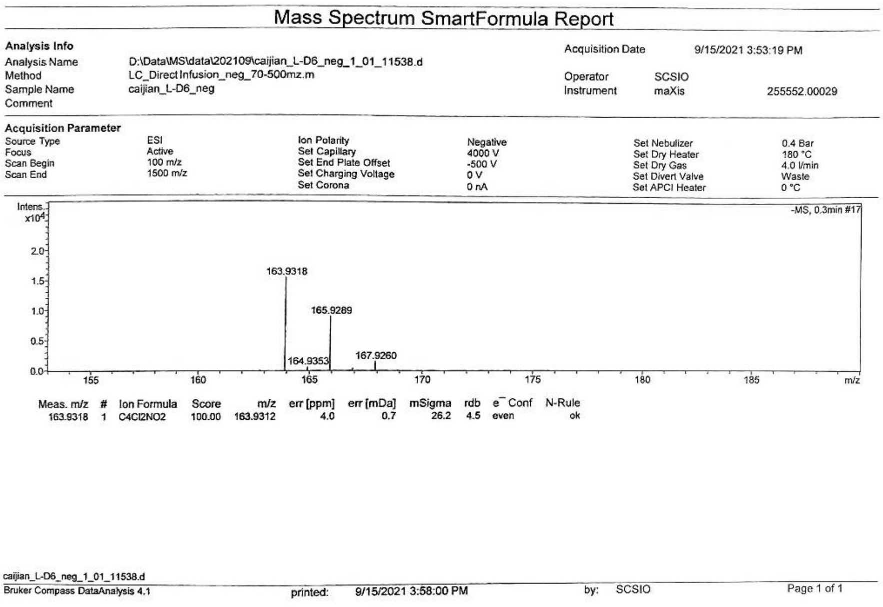

Figure S12: HRESIMS spectrum of **3**.

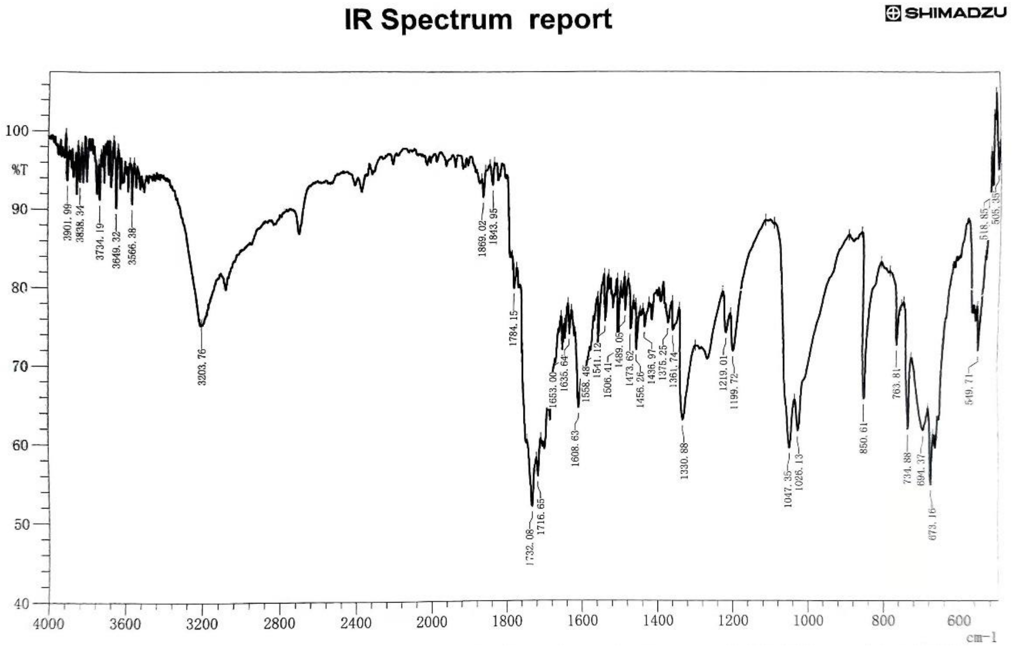

Figure S13: IR spectrum of **3**.

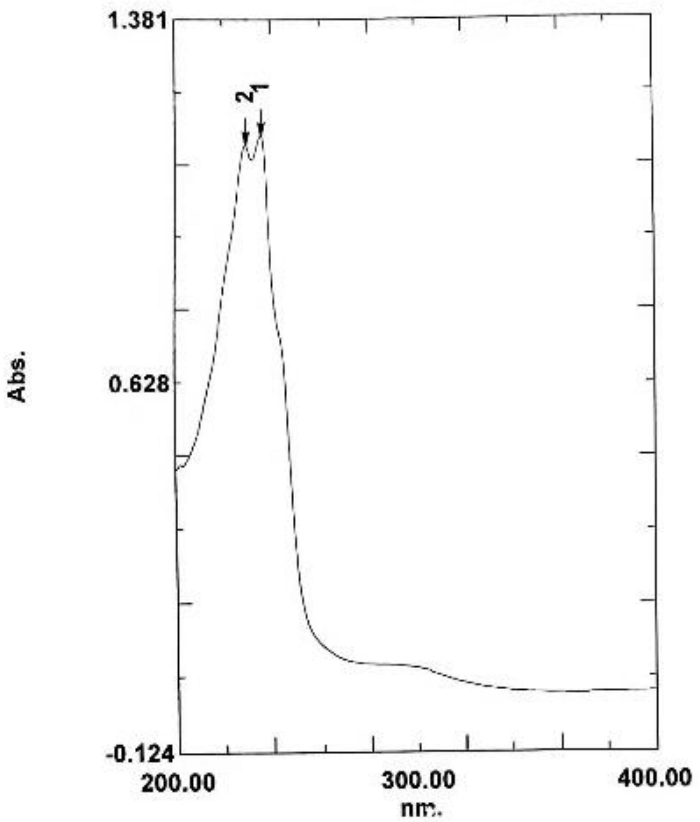

Figure S14: UV spectrum of **3** in MeOH.

ITS sequence of the strain *Mollisia sp*. SCSIO41409

TATTGATATGCTTAAGTTCAGCGGGTATCCCTACTTGATCCGAGGTCAACCTTTAAAAAT
TGGGGGTTTAATAGCAGGACACGCAGACTTAACTGGATTTACACTATCCACCGGCAGG
CTCTGTAGCGAGAAGAAATTACTACGCTTAGGACCCGCAGGTGACGCTACTGGACTCT
AGGGCTGCAAAATTCGCAATCCCCAACACCAAGACAGGCTTGAGTGGTTATAATGACG
CTCGAACAAGCATACCCGGCGGAATACCACCGGGTGCAATGTGCGTTCAAAGATTCGA
TGATTCACTGAATTCTGCAATTCACATTACTTATCGCATTTCGCTGCGTTCTTCATCGATG
CCAGAACCAAGAGATCCGTTGTTGAAAGTTTTAACTATTATATAGTACTCAGACATCAC
TAACATTCAGAGTTTGGTCCTCTGGCAGGCACATGCAGGCAGAGCCCACAGTGGGAG
ACCACGGCCTGCCAAAGCAACAAGAGTATGTAGACACGGATG
